# A New Convenient Method to Assess Antibiotic Resistance and Antimicrobial Efficacy against Pathogenic *Clostridioides difficile* Biofilms

**DOI:** 10.3390/antibiotics13080728

**Published:** 2024-08-03

**Authors:** Lingjun Xu, Bijay Gurung, Chris Gu, Shaohua Wang, Tingyue Gu

**Affiliations:** 1 Department of Chemical & Biomolecular Engineering, Edison Biotechnology Institute, Ohio University, Athens, OH 45701, USA; 2 Department of Biomedical Sciences, Ohio University Heritage College of Osteopathic Medicine, Ohio University, Athens, OH 45071, USA; 3 Infectious and Tropical Disease Institute, Ohio University, Athens, OH 45071, USA

**Keywords:** *Clostridioides difficile*, biocorrosion, antimicrobial, electrochemical test kit, biofilm prevention, biofilm kill

## Abstract

*Clostridioides difficile* is a widely distributed anaerobic pathogen. *C. difficile* infection is a serious problem in healthcare. Its biofilms have been found to exhibit biocorrosivity, albeit very little, but sufficient for it to correlate with biofilm growth/health. This work demonstrated the use of a disposable electrochemical biofilm test kit using two solid-state electrodes (a 304 stainless steel working electrode, and a graphite counter electrode, which also served as the reference electrode) in a 10 mL serum vial. It was found that the *C. difficile* 630∆*erm* Adp-4 mutant had a minimum inhibitory concentration (MIC) for vancomycin twice that of the 630∆*erm* wild type strain in biofilm prevention (2 ppm vs. 1 ppm by mass) on 304 stainless steel. Glutaraldehyde, a commonly used hospital disinfectant, was found ineffective at 2% (*w*/*w*) for the prevention of *C. difficile* 630∆*erm* wild type biofilm formation, while tetrakis(hydroxymethyl)phosphonium sulfate (THPS) disinfectant was very effective at 100 ppm for both biofilm prevention and biofilm killing. These antimicrobial efficacy data were consistent with sessile cell count and biofilm imaging results. Furthermore, the test kit provided additional transient biocide treatment information. It showed that vancomycin killed *C. difficile* 630∆*erm* wild type biofilms in 2 d, while THPS only required minutes.

## 1. Introduction

*Clostridioides difficile* is a Gram-positive and spore-forming anaerobic bacterium widely distributed in the natural environment [1]. *C. difficile* infection (CDI) can lead to a variety of clinical symptoms, including mild to moderate diarrhea and severe colitis, which can be potentially fatal [2,3]. The annual number of CDI cases in United States is estimated to be over 500,000, among which 29,000 deaths occur within 30 days after CDI diagnosis [4,5]. In the United States alone, CDI-associated healthcare costs are estimated at $6.3 billion every year [6]. It has been reported that those aged over 65 years are more vulnerable to CDI [7]. Data in the United States showed that 93% of death cases due to CDI were from adults in this age group [8]. Additionally, older patients were identified to have a higher risk of CDI recurrence [9]. Given the aging population worldwide, CDI has become a significant public health concern [10].

Antibiotics including vancomycin and metronidazole are commonly used for CDI treatment [11], with vancomycin being preferred for severe CDI [12]. Vancomycin impedes peptidoglycan biosynthesis by attaching to D-alanyl-D-alanine at the terminal of the bacterial peptidoglycan precursor, thereby inhibiting cell wall peptidoglycan formation [13]. The subsequent replicating bacteria become vulnerable and weak due to an incomplete and fragile cell wall [14]. In hospital environments, *C. difficile* is also a major threat, as *C. difficile* strains have the ability to produce spores, which contribute to the transmission of CDI [15,16]. Additionally, in the stools of patients with CDI, *C. difficile* spores were also found to exist in abundance [17,18]. Therefore, disinfection and sanitation are necessary to prevent such transmission in hospitals [19]. Glutaraldehyde is a disinfectant widely used in hospitals and agricultural, laboratory, and industrial settings [20,21]. It is a potent peptide cross-linker that reacts with proteins of extracellular polymeric substances (EPS) to disable the amine group on cell wall components [22,23]. It is a good non-oxidizing antimicrobial commonly used to sterilize medical instruments such as endoscopes, according to United States Center for Disease Control and Prevention [24]. Like glutaraldehyde, tetrakis(hydroxymethyl)phosphonium sulfate (THPS) is another readily biodegradable broad-spectrum biocide. It is commonly used in the water treatment industry [25], and among other industries such as oil and gas [26]. It is a non-oxidizing antimicrobial that works as a reducing agent to break disulfide bonds in proteins and enzymes, causing disruption of cell membranes [27,28].

A century ago, microbiologically influenced corrosion or biocorrosion was reported for the first time [29]. Corrosive microorganisms form biofilms on metal surfaces to cause biocorrosion via various mechanisms, including using energetic metals as electron donors in their energy metabolism and secreting corrosive metabolites [30,31,32]. Some pathogenic bacteria were also reported to be culprits in biocorrosion [33]. *Pseudomonas aeruginosa* is involved in cystic fibrosis and other diseases [34,35,36]. It was found to be capable of corroding metals both aerobically and anaerobically as an nitrate-reducing bacterium (NRB) [37,38,39,40]. NRB *P. aeruginosa* can take electrons from metals such as carbon steel or stainless steel and use nitrate as the terminal electron acceptor, which is eventually reduced to nitrogen gas or ammonium, as shown below [41,42].

Fe → Fe^2+^ + 2e^−^ (*E*° = −447 mV_SHE_) (1)

2NO_3_^−^ + 10e^−^ + 12H^+^ → N_2_ + 6H_2_O (*E*°’ = +749 mV_SHE_) (2)

NO_3_^−^ + 8e^−^ + 10H^+^ → NH_4_^+^ + 3H_2_O (*E*°’ = +358 mV_SHE_) (3)

The coupling of iron oxidation with nitrate reduction to N_2_ or NH_4_^+^ leads to a positive cell potential of ∆*E*°’ = +1.20 V or 805 mV, respectively, indicating energy production from the corrosion. This energy can be harvested by the sessile cells in a biofilm [43].

To combat biocorrosion, biocides are commonly used to treat corrosive biofilms [30,44]. Glutaraldehyde and THPS are the most popular green biocides used in treating biofilms to mitigate biocorrosion in industrial applications [45]. Compared to planktonic cells, sessile cells living in biofilms are far more difficult to mitigate owing to several protective mechanisms provided by biofilms [46]. As a result, sessile cells typically require a higher dosage of biocides or other antimicrobial agents to treat [47,48].

Common electrochemical techniques include linear polarization resistance (LPR), electrochemical impedance spectroscopy (EIS), and potentiodynamic polarization (PDP), which is also known as Tafel scan. They apply either direct current (DC) or alternate current (AC) signals to measure corrosion behaviors [49]. Unlike traditional metal coupon weight loss measurement, which gives one-shot results at the end of the experiment period, electrochemical tests possess the advantage of yielding near real-time transient results. In addition, electrochemical tests are very sensitive to detecting corrosion signals, even though the weight loss is negligible, such as corrosion of titanium and high-grade stainless steels [50,51,52]. This means that pathogenic biofilms exhibiting very little corrosivity that have no practical significance in corrosion can be studied using electrochemical methods for their growth/health on a metal surface.

Since the biocorrosivity of a biofilm is closely related to biofilm growth and health, the measured corrosion rate response can be used to monitor biofilm growth/health and antimicrobial efficacy. Effective biofilm treatment is reflected by a large reduction in corrosion rate or a large increase in corrosion resistance [53,54]. This is because biofilms are behind biocorrosion, as they either harvest electrons from energetic metals or secrete corrosive metabolites that have higher local concentrations underneath biofilms, with H_2_ often being a corrosion product [30,55].

*C. difficile* biofilms were recently found to be mildly corrosive against carbon steel and stainless steel [56]. Thus, sensitive electrochemical corrosion measurements can be used to reflect biofilm health. The objective of this work was to prove the hypothesis that an electrochemical biofilm test kit can be conveniently used to assess antimicrobial treatment of *C. difficile* biofilms. Vancomycin, glutaraldehyde, and THPS were assessed in *C. difficile* biofilm treatment. Biofilm prevention tests and biofilm kill tests were both performed.

## 2. Results and Discussion

### 2.1. Weight Loss Analysis

After a 14-d incubation with *C. difficile* 630∆*erm* in a 125 mL vial, 304 SS coupons were cleaned and weighed. The weight loss of the 304 SS coupons was found to be negligible. This is common in stainless steel biocorrosion. Even the super corrosive sulfate-reducing bacterium (SRB) Desulfovibrio ferrophilus IS5 did not cause measurable weight loss of 304 SS in one week [57]. Three replicate X65 carbon steel coupons in a separate vial had a 14-d weight loss of 1.1 ± 0.3 mg/cm^2^, equivalent to a corrosion rate of 0.036 ± 0.010 mm/a. In the abiotic control vial, the weight loss was undetectable. The corrosion rate of *C. difficile* 630∆*erm* against X65 was nearly four times less than that of nitrate-reducing P. aeruginosa against C1018 carbon steel (2.1 mg/cm^2^ in one week) [41]. The weight loss here confirms the mild corrosivity of *C. difficile* 630∆*erm*. This forms the basis for electrochemical assessment of its biofilms.

### 2.2. C. difficile 630∆erm Treatment with Vancomycin

#### 2.2.1. Planktonic and Sessile Cell Enumeration

**Figure 1:**
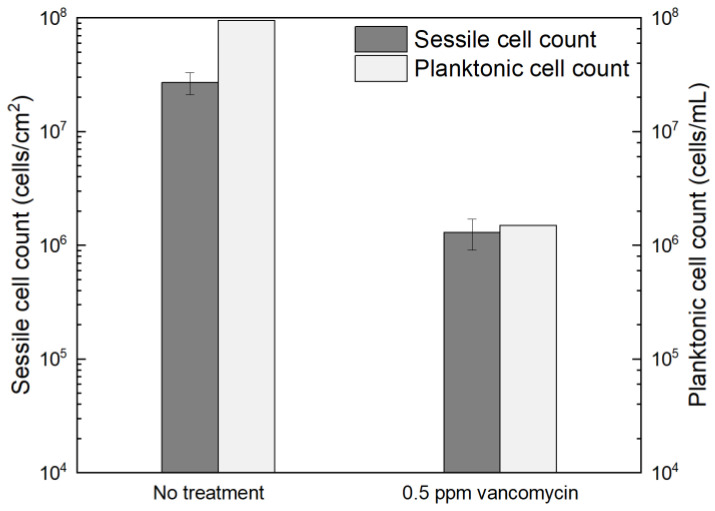
Sessile cell counts on 304 SS coupons and planktonic cell counts after a 7-d incubation at 37 °C with *C. difficile* 630∆*erm* in 125 mL anaerobic vials, each containing 50 mL brain heart infusion supplement (BHIS) medium with and without vancomycin treatment. (Error bars are standard deviations from three replicates in the same vial).

Figure 1 shows the planktonic and sessile cell count results after a 7-d incubation with *C. difficile* 630∆*erm* in 125 mL anaerobic vials. The planktonic cell count was 9.5 × 10^7^ cells/mL, and a (2.7 ± 0.6) × 10^7^ cells/cm^2^ sessile cell count was obtained on the 304 SS coupons. These data suggested healthy *C. difficile* 630∆*erm* growth in the absence of antimicrobial treatment. With 0.5 ppm (*w*/*w*) vancomycin, the planktonic cell count and sessile cell count declined to 1.5 × 10^6^ cells/mL, and (1.3 ± 0.4) × 10^6^ cells/cm^2^, respectively. The planktonic and sessile cell counts were below the hemocytometer detection limit (5 × 10^4^ cells/mL and 5 × 10^4^ cells/cm^2^) with 1 ppm vancomycin. Therefore, 0.5 ppm vancomycin achieved 1.8 log (98.4%) and 1.3 log (95.2%) reductions in *C. difficile* 630∆*erm* planktonic cells and sessile cells, respectively, and 1 ppm vancomycin achieved at least 3.3 log (99.95%) and 2.7 log (99.8%) reductions, respectively. For inhibition of *C. difficile* 630∆*erm* biofilm growth, 0.5 ppm vancomycin was biocidal, while 1 ppm vancomycin was much more effective. Because the minimum inhibitory concentration (MIC) is defined as the dosage needed to inhibit a microbe to an undetectable level [58], the MIC of vancomycin for *C. difficile* 630∆*erm* biofilm prevention was deemed to be 1 ppm for both planktonic and sessile cells.

#### 2.2.2. Confocal Laser Scanning Microscopy (CLSM) Biofilm Imaging

**Figure 2:**
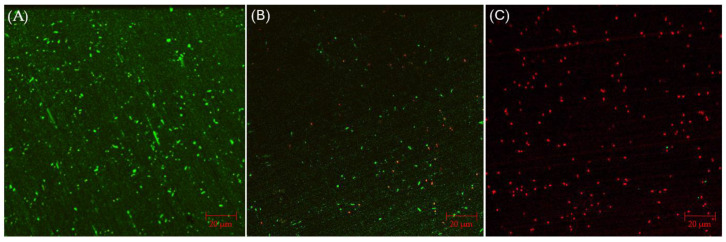
CLSM biofilm images of *C. difficile* 630∆*erm* on 304 SS coupons after a 7-d incubation in 125 mL anaerobic vials with (**A**) no treatment, (**B**) 0.5 ppm vancomycin, and (**C**) 1 ppm vancomycin.

CLSM biofilm images of 304 SS coupons are shown in Figure 2, in which the parallel lines across the entire coupon surfaces were metal polishing lines. Without any antimicrobial, abundant live *C. difficile* 630∆*erm* sessile cells (represented by green dots) were seen on the 304 SS coupon surface, suggesting healthy biofilm formation. The number of live cells was considerably reduced and dead cells (represented by red dots) appeared when 0.5 ppm vancomycin was added. With 1 ppm vancomycin, live *C. difficile* 630∆*erm* cells were rarely seen among abundant dead cells on the 304 SS coupon surface. The CLSM findings are consistent with the sessile cell count results, indicating that 0.5 ppm vancomycin showed some effect in inhibiting *C. difficile* 630∆*erm* biofilm growth but was not sufficient to prevent it, while 1 ppm vancomycin prevented biofilm formation on the 304 SS coupon surface very well.

#### 2.2.3. Electrochemical Biofilm Test Kit Measurements

**Figure 3:**
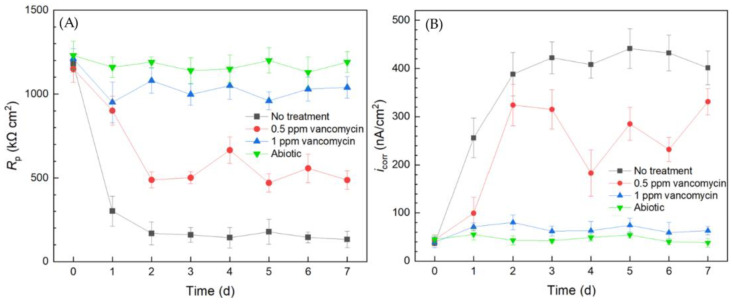
(**A**) Variations of *R*_p_ vs. time from LPR and (**B**) *i*_corr_ vs. time from Tafel scans during 7-d incubation of 304 SS WE with *C. difficile* 630∆*erm* in 10 mL electrochemical cells containing 5 mL BHIS medium with vancomycin added upon inoculation.

Electrochemical tests including LPR and Tafel scans were conducted using 10 mL electrochemical cells. Each LPR scan took only 2 min, while each Tafel scan took 40 min. Polarization resistance (*R*_p_) and corrosion current density (*i*_corr_) were obtained from LPR and Tafel scans, respectively. A higher *R*_p_ or a lower *i*_corr_ corresponds to a lower corrosion rate [49]. In Figure 3A, the abiotic control *R*_p_ curve fluctuated slightly and the *R*_p_ value remained high throughout the 7-d incubation period, suggesting the system was free of biocorrosion rate take-off. For the curve without treatment, *R*_p_ quickly dropped from the initial high value in the first 2 d and stabilized for the remaining incubation time. This *R*_p_ trend reflected biofilm formation on the 304 SS working electrode (WE) surface in the initial 2 d and reached maturity at around 2–3 d. Since the *C. difficile* 630∆*erm* biofilm was responsible for biocorrosion, the corrosion rate (reflected by 1/*R*_p_) increased with biofilm formation, and the corrosion rate leveled off after the biofilm matured. In the presence of 0.5 ppm vancomycin, the *R*_p_ curve also showed an obvious decreasing trend at the beginning and fluctuated afterwards. The 0.5 ppm vancomycin *R*_p_ values were consistently higher than those from the no treatment curve, indicating lower corrosion due to vancomycin. The *R*_p_ curve of 1 ppm vancomycin was close to the abiotic control, suggesting excellent biofilm prevention.

The Tafel scan results in Figure 3B demonstrate an *i*_corr_ sequence (corrosion rate sequence) of no treatment > 0.5 ppm vancomycin > 1 ppm > abiotic, which was in agreement with the 1/*R*_p_ sequence in Figure 3A. The *i*_corr_ values of no treatment and 0.5 ppm vancomycin increased considerably from 0 d to 2 d with biofilm formation on the 304 SS WE surface. The 1 ppm vancomycin *i*_corr_ curve was only slightly higher than the abiotic control, suggesting an excellent biofilm prevention effect. Therefore, the *R*_p_ and *i*_corr_ electrochemical test results both corroborated the cell count and biofilm observation results above. Moreover, the two electrochemical tests provided extra transient information during the 7-d incubation period, unlike the one-shot sessile cell count and biofilm imaging results at the end of the incubation period. It is worth noting that, in Figure 3, the 0 d *R*_p_ and *i*_corr_ values may be viewed as abiotic control values, because they were obtained when biofilm had not built up on the 304 SS WE.

**Figure 4:**
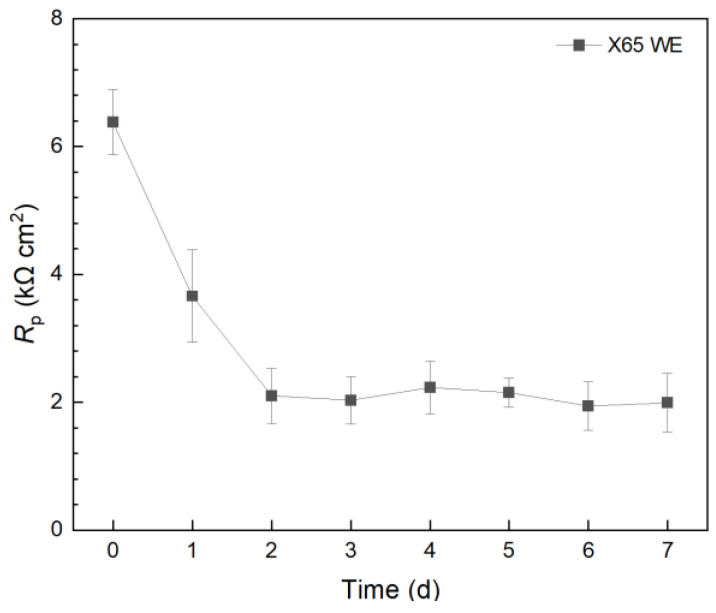
Variation of *R*_p_ vs. time from LPR during 7-d incubation of X65 WE with *C. difficile* 630∆*erm* in 10 mL electrochemical cells containing 5 mL BHIS medium.

In a separate 10 mL electrochemical cell, X65 carbon steel was employed as the WE and incubated with *C. difficile* 630∆*erm*. The 7-d *R*_p_ profile in Figure 4 presented a similar trend to that of the 304 SS WE without treatment. However, the X65 *R*_p_ values were 10^2^ lower than those of the 304 SS WE. Thus, the corrosion rate on 304 SS was expected to be 10^2^ lower than X65 carbon steel. This explains the phenomenon that, when the weight loss of X65 carbon steel coupons was small, the weight loss of 304 SS coupons became completely undetectable, which is a common occurrence in corrosion studies. This also means that, if a pathogenic biofilm’s corrosivity is too low, a carbon steel WE can be used to amplify the corrosion rate signal (e.g., 1/*R*_p_ and *i*_corr_) by 10^2^.

### 2.3. Investigating C. difficile 630∆erm Adp-4 Antibiotic Resistance

#### 2.3.1. Planktonic and Sessile Cell Enumeration with Vancomycin Treatment

**Figure 5:**
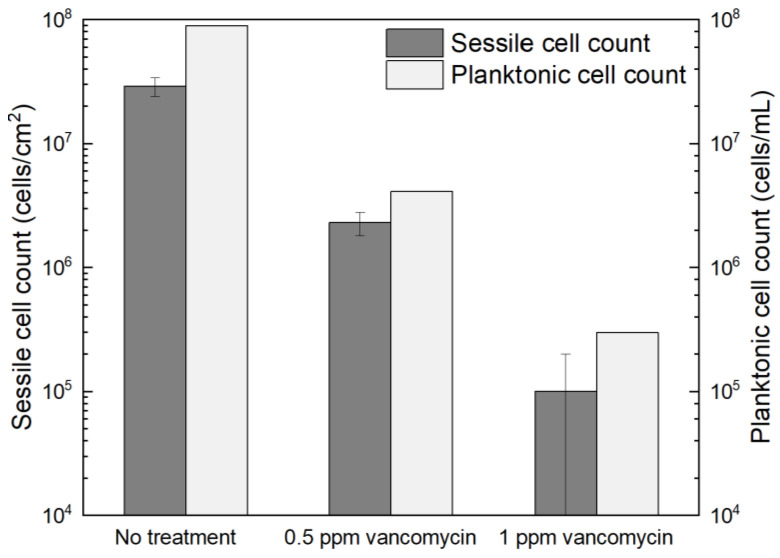
Sessile cell counts on 304 SS coupons and planktonic cell counts after 7-d incubation with *C. difficile* 630∆*erm* Adp-4 in 125 mL anaerobic vials each containing 50 mL BHIS medium with different vancomycin dosages added upon inoculation.

A *C. difficile* mutant 630∆*erm* Adp-4 was incubated in 125 mL anaerobic vials with 304 SS coupons with vancomycin treatment. After the 7-d incubation, the planktonic cell count reached 8.9 × 10^7^ cells/mL, and the sessile cell count on 304 SS coupons was (2.9 ± 0.5) × 10^7^ cells/cm^2^, which were close to those values for the wildtype strain. In Figure 5, 0.5 ppm vancomycin and 1 ppm vancomycin achieved 1.3 log (95.4%) and 2.5 log (99.7%) reductions in planktonic cell count, lowering them to 4.1 × 10^6^ cells/mL and 3.0 × 10^5^ cells/mL planktonic cell counts, respectively. The sessile cell counts on 304 SS coupons were reduced to (2.3 ± 0.5) × 10^6^ cells/cm^2^ and (1.0 ± 1.0) × 10^5^ cells/cm^2^, respectively, equivalent to 1.1 log (92%) and 2.5 log (99.7%) reductions. Compared to *C. difficile* 630∆*erm* wild type, the mutant was more resistant to vancomycin, as 0.5 ppm resulted in less cell reduction and 1 ppm was found to be inadequate to prevent *C. difficile* 630∆*erm* Adp-4 biofilm growth adequately. A higher dosage of 2 ppm vancomycin was tested, leading to both planktonic and sessile cell counts below the aforementioned hemocytometer detection limits, equivalent to at least 3.3 log (99.94%) and 2.8 log (99.8%) reductions in planktonic and sessile cell counts, respectively. Thus, the MIC of vancomycin for *C. difficile* 630∆*erm* Adp-4 was found to be 2 ppm. In studies with very good antimicrobial efficacies, the detection limit (50,000 cells/mL) on a hemocytometer under 400× magnification would fail in measuring the low cell counts, because on average no cell is seen in five separate 0.2 mm × 0.2 mm squares on a hemocytometer. Because CLSM typically uses 400× magnification as well, with a low cell count it is hard to see sessile cells in a magnified view field. Thus, more sensitive cell counting methods such as most probable number (MPN) serial dilution and colony counting using agar plates are used.

#### 2.3.2. CLSM Biofilm Images with Vancomycin Treatment

**Figure 6:**
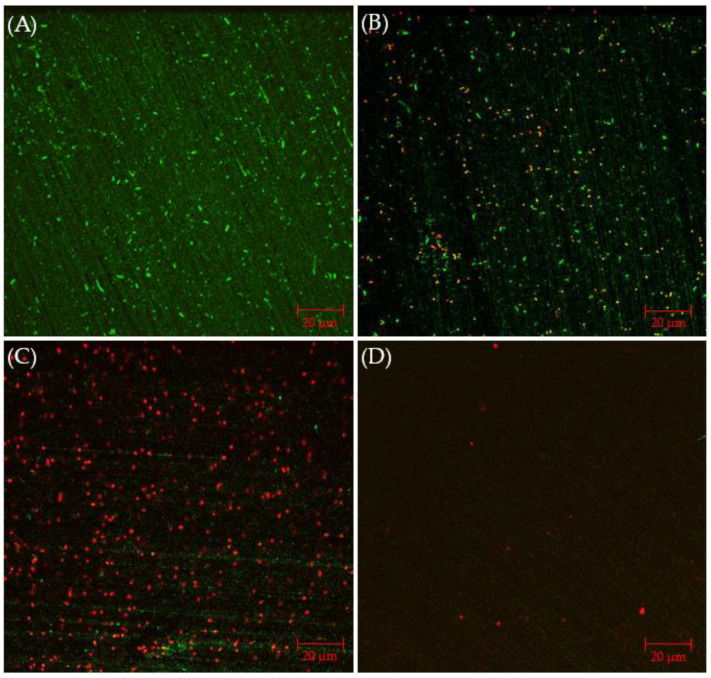
CLSM biofilm images of *C. difficile* 630∆*erm* Adp-4 on 304 SS coupons after 7-d incubation in 125 mL anaerobic vials with (**A**) no treatment, (**B**) 0.5 ppm vancomycin, (**C**) 1 ppm vancomycin, and (**D**) 2 ppm vancomycin.

Figure 6A presents CLSM biofilm images on 304 SS coupons. Under CLSM, a robust biofilm of the *C. difficile* 630∆*erm* Adp-4 mutant was observed on the 304 SS coupon surface. Fewer live cells are seen, with plenty of dead cells, with 0.5 ppm vancomycin in Figure 6B. When the vancomycin dosage increased to 1 ppm, most sessile cells appear dead, but there are still some live cells in Figure 6C. With 2 ppm vancomycin, live cells are not found, and even dead cells are barely seen on the coupon surface in Figure 6D, indicating an excellent biofilm prevention outcome. Thus, the CLSM images confirmed the cell count results in Figure 5, suggesting that 0.5 ppm and 1 ppm vancomycin caused some sessile cell reductions, while 2 ppm vancomycin was deemed to be the MIC dosage for *C. difficile* 630∆*erm* Adp-4. Thus, this mutant was proven to be resistant against vancomycin to a certain degree.

#### 2.3.3. Antibiotic Resistance Investigation Using the Biofilm Test Kit

**Figure 7:**
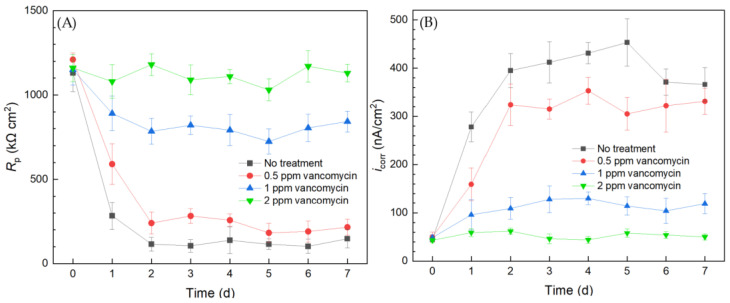
(**A**) Variations of *R*_p_ vs. time from LPR measurement and (**B**) *i*_corr_ vs. time from Tafel scans during 7-d incubation of 304 SS WE with the *C. difficile* 630∆*erm* Adp-4 mutant in 10 mL electrochemical cells containing 5 mL BHIS medium with vancomycin added.

The results from the 10 mL electrochemical biofilm test kit for the *C. difficile* 630∆*erm* Adp-4 mutant with vancomycin treatment for biofilm prevention are presented in Figure 7. The *R*_p_ curve of the no treatment control showed a similar trend to that of the wild type strain, which sharply declined in the initial 2 d and then leveled off. The *R*_p_ curve of the mutant is similar to that of the wild type strain in Figure 3, suggesting similar corrosivity. In Figure 7, 0.5 ppm vancomycin treatment uplifted the *R*_p_ curve. However, its *R*_p_ values were not far from the no treatment control, suggesting insufficient biofilm inhibition. The *R*_p_ curve of 1 ppm vancomycin showed obvious improvement, because *R*_p_ values became considerably higher (i.e., lower corrosivity or biofilm build-up) compared to 0.5 ppm vancomycin treatment. However, compared to the *R*_p_ value at 0 d before the biofilm build-up on the 304 SS WE, the 1 ppm vancomycin *R*_p_ curve in Figure 3A shows an *R*_p_ decline after 0 d, indicating biocorrosivity caused by biofilm build-up. This result means that 1 ppm did not achieve an excellent biofilm prevention effect, unlike 2 ppm vancomycin. Thus, 2 ppm vancomycin is deemed to be the MIC in biofilm prevention for the mutant, which is twice as high as that for the mutant, indicating that the mutant has a level of vancomycin resistance, which was conveniently detected using the electrochemical test kit.

The corrosion rate sequence based on *i*_corr_ results was consistent with the 1/*R*_p_ sequence. The corrosion rates of *C. difficile* 630∆*erm* Adp-4 decreased with higher dosages of vancomycin. The *i*_corr_ curve of 0.5 ppm vancomycin was close to the no treatment control, and they both showed a substantial increase from 0 d to 2 d when biofilms started to become mature. Treatment with 1 ppm vancomycin considerably increased the inhibition effect, as the *i*_corr_ became much lower. However, it did not suppress corrosion well compared to the 0 d *i*_corr_ value when the biofilm had not built up. The *i*_corr_ also suggested that 2 ppm vancomycin is the MIC for biofilm prevention for the mutant. Both the LPR and PDP results indicated that the *C. difficile* mutant had a level of vancomycin resistance compared to the wild type strain. Electrochemical tests were again found to be consistent with cell count and biofilm observation results. Because each LPR scan took only 2 min, while each Tafel scan took 40 min, LPR is the primary choice for electrochemical scans, while Tafel scans can be used as confirmation when the *R*_p_ is not clear-cut.

### 2.4. C. difficile 630∆erm with Disinfectant Treatment for Sanitation

#### 2.4.1. Planktonic and Sessile Cell Enumerations with Disinfectant Treatment

**Figure 8:**
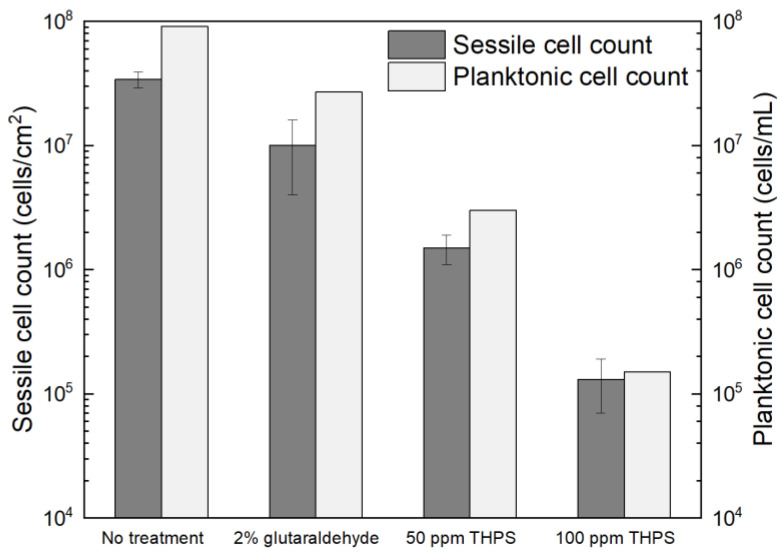
Sessile cell counts on 304 SS and planktonic cell counts after 7-d incubation with *C. difficile* 630∆*erm* in 125 mL anaerobic vials each containing 50 mL BHIS medium with and without biocide.

Glutaraldehyde and THPS were tested in treating *C. difficile* 630∆*erm* biofilms. The dosage of glutaraldehyde used was 2% (*w*/*w*), since 2% (equivalent to 20,000 ppm by mass) glutaraldehyde is included as a disinfectant in the WHO Model Lists of Essential Medicines and was found to be effective in killing different bacterial species [59]. However, after the 7-d incubation with 2% glutaraldehyde, the planktonic cells and sessile cells were reduced from 9.1 × 10^7^ cells/mL and (3.4 ± 0.5) × 10^7^ cells/cm^2^ to 2.7 × 10^7^ cells/mL and (1.0 ± 0.6) × 10^7^ cells/cm^2^, respectively (Figure 8). The 0.5 log (70% and 71%) reductions in two cell counts indicated that the antibiotic effect of 2% glutaraldehyde on *C. difficile* 630∆*erm* biofilms were inadequate. Thus, *C. difficile* 630∆*erm* can be viewed as glutaraldehyde resistant. In comparison, THPS at 50 ppm yielded a planktonic cell count of 3.0 × 10^6^ cells/mL and a sessile cell count of (1.5 ± 0.4) × 10^6^ cells/cm^2^, equivalent to 1.5 log (96.7%) and 1.4 log (95.6%) reductions, respectively. The cell counts were further reduced to 1.5 × 10^5^ cells/mL and (1.3 ± 0.6) × 10^5^ cells/cm^2^, respectively, with 100 ppm THPS. Compared to 50 ppm THPS, 100 ppm THPS achieved extra 1.3 log and 1.1 log reductions or 2.8 log (99.8%) and 2.4 log (99.6%) cumulative reductions in two cell counts, respectively. Therefore, THPS is deemed to be very effective in preventing *C. difficile* 630∆*erm* biofilm formation.

#### 2.4.2. CLSM Biofilm Images with Disinfectant Treatment

**Figure 9:**
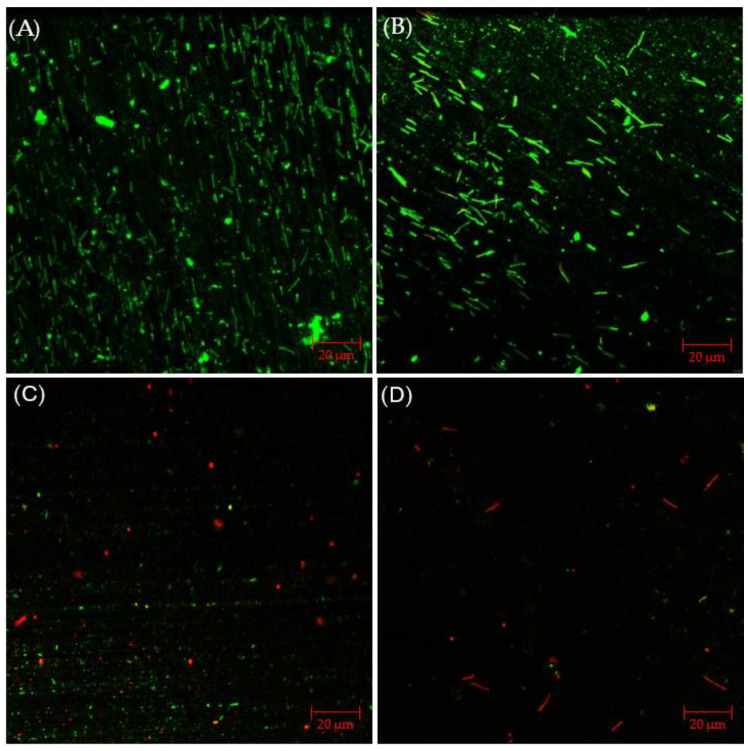
CLSM biofilm images of *C. difficile* 630∆*erm* on 304 SS after 7-d incubation in 125 mL anaerobic vials with (**A**) no treatment, (**B**) 2% glutaraldehyde, (**C**) 50 ppm THPS, and (**D**) 100 ppm THPS.

The CLSM images in Figure 9 illustrated that, with 2% glutaraldehyde, sessile cells observed in *C. difficile* 630∆*erm* biofilm were less dense than those without treatment. However, there were still abundant live cells detected, suggesting inadequate inhibition of *C. difficile* 630∆*erm* biofilm growth. Sessile cells became considerably fewer in the presence of 50 ppm THPS, with both live and dead cells appearing. Nearly all the cells observed were dead in the biofilm treated with 100 ppm THPS, and the total number of cells detected was also further decreased. These biofilm images supported the sessile cell count results.

#### 2.4.3. Assessing Biocide Prevention of *C. difficile* Biofilm Using a 10 mL Biofilm Test Kit

**Figure 10:**
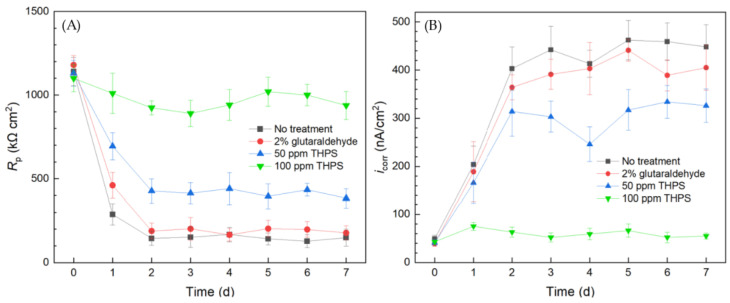
(**A**) Variations of *R*_p_ vs. time from LPR measurement and (**B**) *i*_corr_ vs. time from Tafel scans during the 7-d incubation of 304 SS WE with *C. difficile* 630∆*erm* in 10 mL electrochemical cell (biofilm test kit) containing 5 mL BHIS medium treated with biocide.

The overall 1/*R*_p_ and *i*_corr_ trends in Figure 10 obtained using the 10 mL biofilm test kit both reveal the same corrosion sequence of no treatment > 2% glutaraldehyde > 50 ppm THPS > 100 ppm THPS. Compared to the no treatment control curves, the 2% glutaraldehyde only exhibited slightly higher *R*_p_ and lower *i*_corr_, indicate slight corrosion suppression, which means the inhibition effect of 2% glutaraldehyde on the *C. difficile* 630∆*erm* biofilm was inadequate. In comparison, the *R*_p_ curve of 50 ppm THPS was considerably elevated, and the *i*_corr_ curve was much lower, than the no treatment control. The strong inhibition effect of 50 ppm THPS was thus manifested. For the no treatment control, 2% glutaraldehyde, and 50 ppm THPS, apparent *R*_p_ decreasing and *i*_corr_ increasing trends were seem in the initial two days, corresponding to biofilm formation and maturity. The *R*_p_ of 100 ppm THPS-only treatment slightly declined, with *i*_corr_ remained at a low level, indicating its excellent effectiveness in inhibiting *C. difficile* 630∆*erm* biofilm growth and its biocorrosion.

Thus, the simple and convenient test using the biofilm test kit proved that *C. difficile* 630∆*erm* was resistant to 2% (equivalent to 20,000 ppm by mass). In biocorrosion mitigation, 100 ppm glutaraldehyde was already effective in mitigating biocorrosion caused by much more corrosive SRB [60], just like THPS [61]. Although 100 ppm THPS showed excellent efficacy in *C. difficile* 630∆*erm* biofilm prevention, 2% glutaraldehyde failed. This observation is rather surprising and important in sanitation, because glutaraldehyde is a widely used hospital disinfectant [21,62]. The 10 mL electrochemical biofilm test kit results were consistently in agreement with sessile cell count and biofilm observation outcomes. Thus, electrochemical tests used for biocorrosion analysis were proven to be a useful tool in assessing biofilm growth/health, as long as the biofilm shows at least a low degree of corrosivity that can be detected.

### 2.5. Injection Tests to Kill Pre-Established C. difficile 630∆erm Biofilm Using 10 mL Biofilm Test Kit

Two types of antimicrobial treatment tests are often conducted in lab tests. In biofilm prevention (biofilm inhibition) tests, the antimicrobial agents are added upon microbial inoculation to prevent biofilm formation [63]. The other test, namely the biofilm kill (biofilm eradication) test, is performed by allowing a mature biofilm to form on a surface before injecting antimicrobials. Biofilm killing is usually more demanding than biofilm prevention, requiring a higher dosage of the antimicrobial agents [64,65,66].

**Figure 11:**
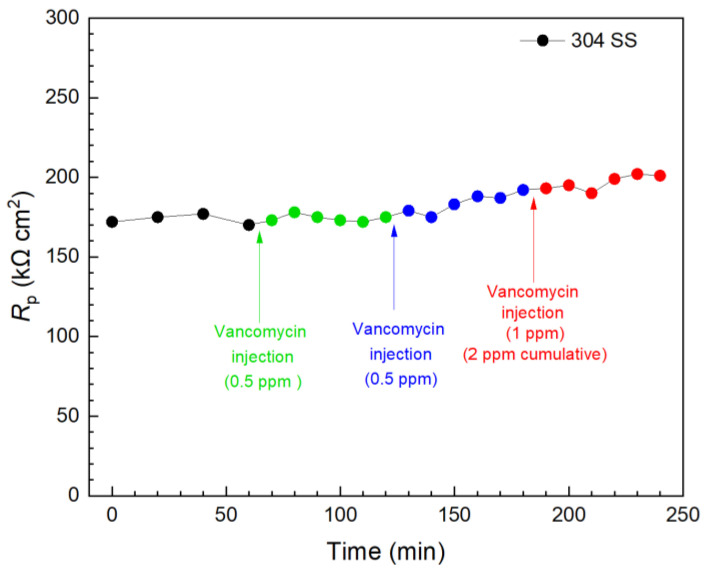
Variations of 304 SS *R*_p_ after tandem injections of vancomycin at 3 d of incubation with *C. difficile* 630∆*erm* in a 10 mL electrochemical cell containing 5 mL BHIS medium. (0 min means 3 d of incubation).

Vancomycin and THPS were found to be effective in inhibiting *C. difficile* 630∆*erm* biofilm build-up on 304 SS above. Thus, they were further evaluated in biofilm kill tests with a 3 d incubation of *C. difficile* 630∆*erm* when the biofilms on 304 SS WE surfaces had already reached maturity, as indicated in Figure 3. In Figure 11, the first injection of 0.5 ppm (concentration in the broth achieved by the injection) vancomycin did not result in a significant *R*_p_ elevation within 1 h, indicating an inadequate kill effect on the pre-established biofilm in this time frame. Another 0.5 ppm vancomycin injection followed, and the total vancomycin concentration in the broth reached 1 ppm, after which a slight increase was observed in 1 h. Biofilm killing is more demanding than biofilm prevention; after all, EPS alone would hinder antimicrobial penetration [67]. In the biofilm prevention test, the MIC of vancomycin was found to be 1 ppm for wild type *C. difficile*. Thus, a subsequent third injection of 1 ppm vancomycin was made 2 h after the first injection, and the vancomycin in broth reached 2 ppm cumulatively after the three injections. A small increase trend was observed, and at 240 min (3 h after the first injection), the overall *R*_p_ increase was found to be 17% (equivalent to corrosion inhibition efficiency). LPR measurements were continued. At 1 d and 2 d after the injections, the overall *R*_p_ increase reached 42% and 49%, respectively. The results showed that vancomycin was effective in eradicating *C. difficile* 630∆*erm*; however, the full kill efficacy time was in days rather than in hours.

**Figure 12:**
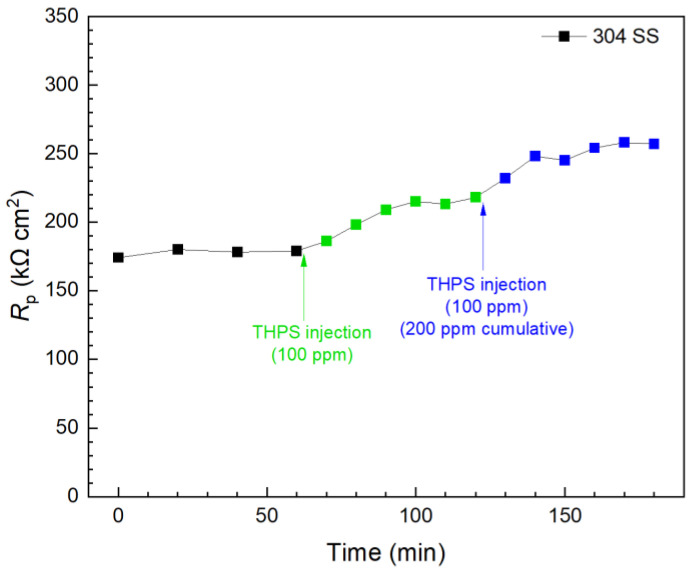
*R*_p_ response to tandem THPS injections at 3 d of incubation with *C. difficile* 630∆*erm* in a 10 mL electrochemical biofilm test kit containing 5 mL BHIS medium.

In contrast, the first 100 ppm THPS injection in another biofilm test kit vial presented in Figure 12 caused an immediate *R*_p_ increase, and the *R*_p_ curve leveled off 30 min after injection. This means 100 ppm THPS achieved its full efficacy within only minutes. Compared to the initial *R*_p_ value (at 0 min), the 100 ppm THPS injection led to a 20% increase after 30 min, suggesting a 20% corrosion inhibition efficiency. The second 100 ppm THPS injection caused another *R*_p_ increase within minutes. With the two injections combined to reach a 200 ppm THPS cumulative dosage in the broth, the overall *R*_p_ increase was 44% (corrosion inhibition efficiency 44%) within 120 min. Thus, THPS was proved to be an effective biocide to kill pre-established *C. difficile* 630∆*erm* biofilms with an efficacy of 44% and a kill time measured in minutes, which was much faster than vancomycin.

**Figure 13:**
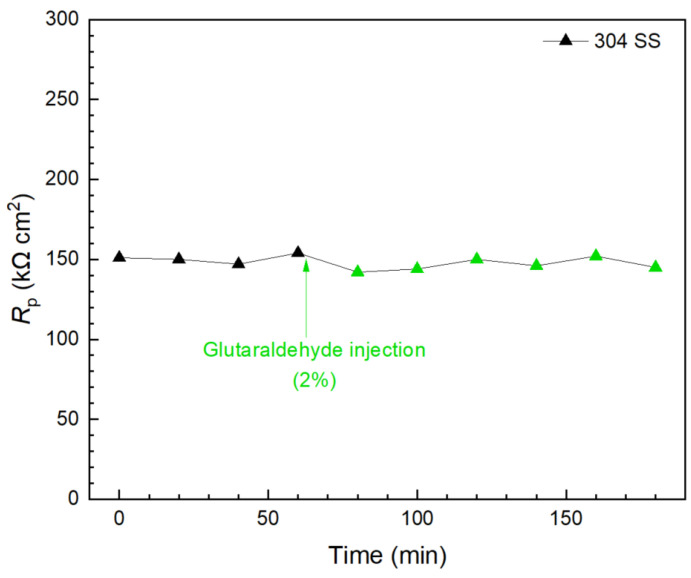
*R*_p_ response to glutaraldehyde (2% in broth) injection at 3 d of incubation with *C. difficile* 630∆*erm* in a 10 mL electrochemical biofilm test kit containing 5 mL BHIS medium.

Glutaraldehyde injection in a separate biofilm test kit vial again shows that, although 2% of glutaraldehyde is the concentration commonly used for disinfection [59,68], it did not show a detectable effect in killing 3 d old *C. difficile* 630∆*erm* on 304 SS because the *R*_p_ curve remained stable 2 h after the injection, as shown in Figure 13. This result is consistent with findings in the prevention test. Apart from biofilm inhibition testing, Figure 11, Figure 12 and Figure 13 demonstrated that the 10 mL electrochemical biofilm test kit was also found to be useful in evaluating biofilm kill efficacy and kill time.

## 3. Materials and Methods

### 3.1. Metals, Bacteria, and Chemicals

We used 304 SS coupons to provide a surface for biofilm attachment. This surgical grade SS is routinely utilized for medical biofilms to build up in investigations [69,70]. X65 carbon steel coupons were also used to provide visual evidence for *C. difficile* biocorrosion, as it was impossible for 304 SS to show corrosion in a short-term lab test. The elemental compositions of 304 SS and X65 carbon steel are listed in Table 1. An inert liquid epoxy coating (3M product 323) was used to protect all surfaces of test coupons except for the top working surface (1 cm × 1 cm). All coupons were polished with 600 grit and sterilized with anhydrous isopropanol before testing. Two different *C. difficile* strains (wild type 630∆*erm* and mutant 630∆*erm* Adp-4) were both cultured in brain heart infusion supplement (BHIS) medium at 37 ℃ (optimal for growth) [71]. Strain *C. difficile* 630∆*erm* is an erythromycin-sensitive and laboratory-generated derivative of the original patient-isolated strain 630 and is commonly used by *C. difficile* researchers as a reference strain for generating mutants (PMID: 33658275). *C. difficile* 630∆*erm* Adp-4 is an adaptive mutant based on *C. difficile* 630∆*erm*.

The initial pH of the BHIS medium was adjusted to 7.0 using 5% HCl (*w*/*w*). The medium was autoclave-sterilized and then sparged with filter-sterilized N_2_ for 1 h to remove dissolved oxygen. An N_2_-filled anaerobic chamber was employed for the anaerobic manipulations, where *C. difficile* was inoculated with the test coupons in deoxygenated BHIS medium (with 1:100 of inoculum to culture medium volumetric ratio). The chemicals used in this work were all purchased from Fisher Scientific (Pittsburgh, PA, USA) or Sigma-Aldrich (St. Louis, MO, USA).

### 3.2. Weight Loss Measurement

To confirm the corrosivity of *C. difficile* 630∆*erm*, it was incubated with 304 SS coupons in 125 mL anaerobic vials containing 50 mL BHIS medium. However, for such a weakly corrosive microbe, it was expected to cause no weight loss on corrosion-resistant 304 SS. Therefore, X65 carbon steel, which is far more prone to biocorrosion, was also employed in the weight loss test. X65 carbon steel coupons were incubated in separate anaerobic vials for 14 d at 37 °C. After the 14 d incubation, 304 SS and X65 carbon steel coupons were retrieved from their respective anaerobic vials and cleaned with freshly prepared Clarke’s solution for 30 s to remove corrosion products and biofilms on the coupon surfaces following the ASTM G1–03 protocol before weighing [72].

### 3.3. Planktonic and Sessile Cell Counts

First, 304 SS coupons were incubated with *C. difficile* in different anaerobic vials with different antimicrobial treatments. After a 7-d incubation, motile planktonic cells in each vial were first counted on a hemocytometer. The planktonic cell samples on the hemocytometer were observed under an optical microscope at 400× magnification [57]. For sessile cell counting, 304 SS coupons were first taken out from each vial after the 7-d incubation. A pH 7.4 phosphate-buffered saline (PBS) solution was used to rinse the coupon surfaces to remove planktonic cells. After that, sessile cells were collected from coupon surfaces with a sterile brush-like applicator into 10 mL (or 1 mL if sessile cell count was low due to antimicrobial treatment) PBS solution. Then, the applicator, the coupon, and the PBS solution were vortexed together for 30 s in a 50 mL centrifuge tube to obtain an evenly distributed cell suspension. The cell suspension was also enumerated on a hemocytometer.

### 3.4. CLSM Biofilm Observation

After the 7-d incubation, the biofilm on each coupon surface was visualized at 400× magnification under a CLSM machine (Model LSM 510, Carl Zeiss, Jena, Germany). Coupons with different antimicrobial treatments were rinsed in a pH 7.4 PBS solution right after being retrieved from the anaerobic vials to remove culture medium and loosely attached planktonic cells. Biofilms were then stained with a Live/Dead^®^ BacLight™ Bacterial Viability Kit L7012 (Life Technologies, Grand Island, NY, USA) [73]. After staining, live cells and dead cells were detected as green dots and red dots, respectively, under CLSM.

### 3.5. Electrochemical Tests in Biofilm Test Kit (10 mL Electrochemical Cell)

**Figure 14:**
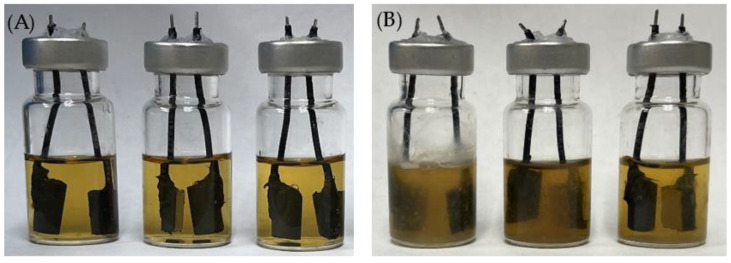
Electrochemical glass cells before (**A**) and after (**B**) 7-d incubation of *C. difficile* 630∆*erm* in 5 mL BHIS medium with (left to right) no treatment, 0.5 ppm vancomycin, and 1 ppm vancomycin at 37 °C.

In all the electrochemical tests, a 10 mL miniature electrochemical cell design (electrochemical test kit) was adopted. Each 10 mL electrochemical cell (Figure 14) contained a 304 SS (1 cm × 1 cm) WE and a graphite rod (0.64 cm diameter and 1 cm height) counter electrode/pseudo-reference electrode (CE/p-RE). This disposable electrochemical test kit design required only two solid-state electrodes in a standard 10 mL serum vial. Each electrochemical cell was pre-filled with N_2_ gas. It was injected with 5 mL BHIS medium with different antimicrobial treatments. The injection syringe’s plunger was retracted at the end of the injection to remove approximately 5 mL N_2_. A PCI4/750 potentiostat (Gamry Instruments, Inc., Warminster, PA, USA) was employed to carry out all the electrochemical scans. During the 7-d incubation tests, LPR and PDP scans were performed on a daily basis. The LPR scan was carried out with a 0.167 mV/s scan rate ranging from −10 mV to 10 mV vs. open circuit potential (OCP). PDP scans were conducted using dual-half scans at a scan rate of 0.167 mV/s on the same WE from 0 mV to −200 mV (vs. OCP) and 0 mV to +200 mV (vs. OCP) [74].

In biofilm prevention testing, an antimicrobial was added to the culture medium upon inoculation. Electrochemical measurements were made daily, starting from 0 d. Injections in biofilm kill testing were performed after 3 d of incubation, when mature biofilms were already formed on the WE surfaces. LPR was first scanned in each electrochemical cell to wait for a stable *R*_p_ curve within 1 h. After that, vancomycin, glutaraldehyde, and THPS at different concentrations were injected into separate 10 mL electrochemical cells. Each electrochemical cell was gently shaken for 3 min after injection to disperse the injected chemicals evenly. The LPR scan was then continued until the *R*_p_ curve stabilized.

## 4. Conclusions

This work demonstrated the use of a disposable biofilm test kit (a 10 mL electrochemical cell with two solid-state electrodes) that conveniently detected *C. difficile* 630∆*erm* biofilm growth on 304 SS and its biofilm prevention and biofilm kill efficacies using vancomycin, glutaraldehyde, and THPS. It was found that the MIC for biofilm prevention was 1 ppm for vancomycin and 100 ppm for THPS. Surprisingly, 2% glutaraldehyde was not effective. In the biofilm kill test, both 1 ppm vancomycin and 100 ppm THPS were effective. The biofilm test kit also found that the *C. difficile* 630∆*erm* Adp-4 mutant had some vancomycin resistance, requiring MIC of 2 ppm (vs. 1 ppm for the wild type) in biofilm prevention. These test results were consistent with the sessile cell count and CLSM biofilm image results. Furthermore, the electrochemical test kit provided daily biofilm growth information reflected by corrosivity, while the cell count and CLSM results were cumulative one-shot results. The injection test results from the biofilm test kit indicated that the vancomycin kill time was 2 d, while THPS took only minutes. The results in this work suggest that the biofilm test kit is a powerful tool to study antibiotic resistance and antimicrobial efficacy against pathogenic biofilms, as long as they exhibit at least very low corrosivity. Because a microbial sample only needs to be injected in a sealed serum vial without subsequent exposure during electrochemical scans, the disposable test kit is safer for handling pathogenic samples compared to traditional microplates with wells. It is also field-use friendly because only a potentiostat instrument is needed. Portable potentiostats are readily available commercially.

## Figures and Tables

**Table 1:** Elemental compositions (wt. %) of 304 SS and X65 carbon steel (Fe balance).

Metal	C	P	S	N	Si	Mn	Cr	Ni	Cu	Mo	V	Nb	Ti
304 SS	0.063	0.031	0.002	0.045	0.312	1.42	18.3	8.12	0.316	0.297			
X65	0.16	0.02	0.01		0.45	1.65					0.09	0.05	0.06

## Data Availability

The data presented in this work are available on request from the corresponding author.
